# Supplementary material for: Impacts of stress and aging on spore health in Schizosaccharomyces pombe
Source: bioRxiv. 2025 May 30:2025.05.29.656811. Preprint. [Version 1] doi: 10.1101/2025.05.29.656811 (PMC12154735; doi:10.1101/2025.05.29.656811)
Supplement: 1 [file NIHPP2025.05.29.656811V1-supplement-1.pdf]

bioRxiv preprint doi: https://doi.org/10.1101/2025.05.29.656811; this version posted May 30, 2025. The copyright holder for this preprint (which was not certified by peer review) is the author/funder, who has granted bioRxiv a license to display the preprint in perpetuity. It is made available under aCC-BY 4.0 International license.

Figure S1. Storage temperature affects spore health and longevity. (A) Cartoon of the spore isolation approach used for all experiments (see methods for more details). A haploid strain proficient in mating-type switching (h90) was cultured in rich media (YES) then plated on sporulation media (MEA) at 25°C for 9 days. The spores were then scraped into tubes and the remaining vegetative cells were killed with glusulase and ethanol treatment. (B) One population of spores stored in water was split and stored at 4°C, 25°C, 32°C, or 37°C. The stored spores were then sampled over time to assay the number of colony-forming units (CFUs) on rich media (YEAS), which were normalized to the time zero starting point. The average of 3 replicates is shown and the error bars represent standard deviations. ** indicates p value < 0.01, comparing areas under the curves via t-test. These are the same data as shown in Figure 1A with more timepoints displayed. (C) This plot follows the same format as (B), but shows the variability present within and between experiments. Batch 1 (orange shades) is a distinct experiment from that shown in (B) and each of three replicates of spores stored at 4°C (dotted lines) and 25°C (solid lines) is shown. Batch 2 (blue shades) is the same data as shown in (B) for spores stored at 4°C (dotted lines) and 25°C (solid lines), except each individual replicate is plotted. (D) An independent, but analogous experiment as that presented in (B) and (C) where the viability of spores stored at 25°C, 37°C, 42°C, and 55°C was assayed over time in two batches. (E) To

bioRxiv preprint doi: https://doi.org/10.1101/2025.05.29.656811; this version posted May 30, 2025. The copyright holder for this preprint (which was not certified by peer review) is the author/funder, who has granted bioRxiv a license to display the preprint in perpetuity. It is made available under aCC-BY 4.0 International license.

prepare cells for imaging via timelapse microscopy experiments in this study, we placed spores on agarose plates (YEAS or PMG). We then took a punch of this agar plate and transferred the agar plug to an 8-well Ibidi dish, placing it upside down, trapping the spores between the plug and dish.

Figure S2. Storage temperature affects spore health. (A-E) Imaging analyses of spores stored at 4°C for one day or 405 days before being plated on rich media (YEAS) to induce germination at 32°C. Cells were imaged every 10 minutes for 1000 minutes. (A) Timelapse microscopy images of germinating spores. The time in minutes is displayed and the scale bars represent 10 µm. (B) Cell area (µm^2) and (C) cell aspect ratio (AR) during germination. The shaded area around each line represents standard error. In some instances, the standard error is thinner than the line. Note that these cell areas provided by the deep learning algorithm are slightly larger than the actual cell areas (see methods). (D) The fraction of spores manually observed to germinate by the end of the 1,000-minute timelapse. N>50 spores per sample. ** indicates p-value < 0.01 via Fisher's exact test. (E) Time of first division (when the AR of a cell exceeded 3). ** indicates p-value <0.01 via ANOVA test. N>100 spores per sample. The experiments in (F-J) duplicate those shown in (A-E), but the spores were stored for 15 days or 397 days.

Figure S3. Storage temperature affects spore health. Imaging analyses of spores stored at 4°C for one, 65, or 438 days before being plated on media to induce germination at 32°C. Cells were imaged every 10 minutes for 1,400 minutes. (A) Timelapse microscopy images of spores germinating on rich media (YEAS, left) and synthetic media (PMG, right). The time in minutes is displayed and the scale bars represent 10 µm. The 438-day-old sample on YEAS lost focus at the 650-minute time frame (see methods for details on how such samples were scored). (B) Cell area (µm^2) and (C) cell aspect ratio (AR) during germination on rich media (YEAS; solid lines)

bioRxiv preprint doi: https://doi.org/10.1101/2025.05.29.656811; this version posted May 30, 2025. The copyright holder for this preprint (which was not certified by peer review) is the author/funder, who has granted bioRxiv a license to display the preprint in perpetuity. It is made available under aCC-BY 4.0 International license.

and synthetic media (PMG; dotted lines). The shaded area around each line represents standard error. In some instances, the standard error is thinner than the line. Note that these cell areas provided by the deep learning algorithm are slightly larger than the actual cell areas (see methods). (D) The fraction of spores manually observed to germinate on YEAS and PMG by the end of the 1,400-minute timelapse. N>50 spores per sample. ** indicates p-value < 0.01, Fisher's exact test. (E) Time of first division (when the AR of a cell exceeded 3) on YEAS and PMG. ** indicates p-value <0.01 via ANOVA test. N>100 spores per sample.

Figure S4. Storage temperature affects spore health. The data in A-B are the same data as those shown in Figure 1C-D but are plotted differently (individual data points rather than population averages) to more clearly illustrate how cell aspect ratio and area change over the course of germination. (A) Aspect Ratio (AR) over time of spores germinating on rich media (YEAS) at 32°C after previous storage of 3 days at 25°C or 42°C in water. (B) Two-dimensional histograms showing the AR and cell area of the entire population of spores analyzed in panel (A). Contour lines were added to help visualize concentrations of cells. (C) The starting area (μm^2) of all spores (left) or of only the successfully germinated spores analyzed in panel (A). N>50 spores per sample. ** indicates p-value < 0.01 via ANOVA test.

Figure S5. Storage temperature affects spore health. Imaging analyses of spores stored for three days at 25°C or 42°C before being plated on media to induce germination at 32°C. Cells were imaged every 10 minutes for 1,750 minutes. (A) Timelapse microscopy of spores germinating on rich media (YEAS, left) and synthetic media (PMG, right). The yellow arrows illustrated an elongated germination phenotype. The time in minutes is displayed and the scale bars represent 10 μm. (B) Cell area (μm^2) and (C) cell aspect ratio (AR) during germination on rich media (YEAS; solid lines) and synthetic media (PMG; dotted lines). The shaded area around each line represents standard error. In some instances, the standard error is thinner

bioRxiv preprint doi: https://doi.org/10.1101/2025.05.29.656811; this version posted May 30, 2025. The copyright holder for this preprint (which was not certified by peer review) is the author/funder, who has granted bioRxiv a license to display the preprint in perpetuity. It is made available under aCC-BY 4.0 International license.

than the line. Note that these cell areas provided by the deep learning algorithm are slightly larger than the actual cell areas (see methods). (D) The fraction of spores manually observed to germinate on YEAS and PMG by the end of the 1,750-minute timelapse. N>50 spores per sample. ** indicates p-value < 0.01 via Fisher's exact test. (E) The percentage of spores displaying the indicated outgrowth phenotypes on YEAS and PMG. N>50 outgrowths per sample. ** indicates p-value < 0.01 via Fisher's exact test. (F) Time of first division (when the AR of a cell exceeded 3) on YEAS and PMG. ** indicates p-value <0.01 via ANOVA test. N>100 spores per sample. (G) Starting area (μm^2) of all (left) or of only the successfully germinated (right) spores on YEAS and PMG. N>50 spores per sample. ** indicates p-value < 0.01 via ANOVA test. (H) Average time of first division and (I) Average germination efficiency of spores stored for 2-4 days at 25°C (N=6) or 42°C (N=6) across all timelapses with various germination temperatures and media.

Figure S6. Storage temperature affects spore health. Imaging analyses of spores stored for 28 days at 25°C or 37°C before germination was induced at 32°C on rich media (YEAS). Cells were imaged every 10 minutes for 1,200 minutes. (A) Timelapse microscopy images of germinating spores. The time in minutes is displayed and the scale bars represent 10 μm. (B) Cell area (μm^2) and (C) cell aspect ratio (AR) during germination. The shaded area around each line represents standard error. In some instances, the standard error is thinner than the line. Note that these cell areas provided by the deep learning algorithm are slightly larger than the actual cell areas (see methods). (D) The fraction of spores manually observed to germinate by the end of the 1,200-minute timelapse. N>50 spores per sample. ** indicates p-value < 0.01, Fisher's exact test. (E) The percentage of spores displaying the indicated outgrowth phenotypes. N>50 outgrowths per sample. ** indicates p-value < 0.01, Fisher's exact test. (F) Time of first division (when the AR of a cell exceeded 3). ** indicates p-value <0.01 via ANOVA

bioRxiv preprint doi: https://doi.org/10.1101/2025.05.29.656811; this version posted May 30, 2025. The copyright holder for this preprint (which was not certified by peer review) is the author/funder, who has granted bioRxiv a license to display the preprint in perpetuity. It is made available under aCC-BY 4.0 International license.

test. N>100 spores per sample. (G) Starting area (μm^2) of all (left) or of only successfully germinated (right) spores. N>50 spores per sample. ** indicates p-value < 0.01 via ANOVA test.

Figure S7. Storage temperature affects spore health. Imaging analyses of spores stored 28 days at 25°C or 37°C before germination was induced on rich media (YEAS) or synthetic media (PMG) at 32°C. The cells were imaged every 10 minutes for 3,000 minutes. (A) Timelapse microscopy images of cells during germination. The storage temperature and germination medium are listed to the right of each row of images. The time in minutes is displayed and the scale bars represent 10 μm. (B) Cell area (μm^2) and (C) cell aspect ratio (AR) during germination. The shaded area around each line represents standard error. In some instances, the standard error is thinner than the line. Note that these cell areas provided by the deep learning algorithm are slightly larger than the actual cell areas (see methods). (D) The fraction of spores manually observed to germinate by the end of the 3000-minute timelapse. N>50 spores per sample. ** indicates p-value < 0.01 via Fisher's exact test. (F) Time of first division (when the AR of a cell exceeded 3). ** indicates p-value <0.01 via ANOVA test. N>100 spores per sample. (E) The percentage of spores displaying the indicated outgrowth phenotypes. N>50 outgrowths per sample. ** indicates p-value < 0.01 via Fisher's exact test.

Figure S8. Storage temperature affects spore health. Imaging analyses of spores stored for 15 days at 25°C or 37°C before germination was induced at 37°C on rich media (YEAS). Cells were imaged every 10 minutes for 1,200 minutes. (A) Timelapse microscopy images of germinating spores. The time in minutes is displayed and the scale bars represent 10 μm. (B) Cell area (μm^2) and (C) cell aspect ratio (AR) during germination. The shaded area around each line represents standard error. In some instances, the standard error is thinner than the line. Note that these cell areas provided by the deep learning algorithm are slightly larger than the actual cell areas (see methods). (D) The fraction of spores manually observed to germinate by

bioRxiv preprint doi: https://doi.org/10.1101/2025.05.29.656811; this version posted May 30, 2025. The copyright holder for this preprint (which was not certified by peer review) is the author/funder, who has granted bioRxiv a license to display the preprint in perpetuity. It is made available under aCC-BY 4.0 International license.

the end of the 1,200-minute timelapse. N>50 spores per sample. ** indicates p-value < 0.01 via Fisher's exact test. (E) The percentage of spores displaying the indicated outgrowth phenotypes. N>50 outgrowths per sample. ** indicates p-value < 0.01 via Fisher's exact test. (F) Time of first division (when the AR of a cell exceeded 3). ** indicates p-value <0.01 via ANOVA test. N>100 spores per sample. (G) Average time of first division and (I) Average germination efficiency of spores stored for 15-35 days at 25°C (N=9) or 37°C (N=4) across all timelapses with various germination temperatures and media.

Figure S9. Storage temperature affects spore health. (A) Scanning electron microscopy images of spores stored for 4 days at 25°C (top), 4 days at 42°C (middle), and 243 days at 25°C (bottom). The inset on the 243-day old sample centers on a 'deflated' spore. Scale bars represent 1 μm. (B) Percentage of deflated and round spores observed in samples represented in panel (A). N> 50 spores. ** indicates p-value < 0.01 via Fisher's exact test. (C) Scanning transmission electron microscopy images of spores stored for 3 days at 25°C (top), 3 days at 42°C (middle), or 270 days at 25°C (bottom). Each of the insets focus on a 'dark-staining' spore, but a 'light-staining' spore is just to the left of the inset spore in the 42°C sample. Scale bars represent 1 μm. (D) Percentage of spores stored for 3 days at 25°C or 42°C, or 270 days at 25°C with light-staining or dark-staining cytoplasm. N> 50 spores. ** indicates p-value < 0.01 via Fisher's exact test.

Figure S10. Storage media significantly affects spore longevity, but not health. (A) One population of spores was split and resuspended in either 0.5% yeast extract solution without glucose (YE) or water and then stored at 4°C. Panel (B) shows a repeat of the experiment shown in (A). In both experiments, the stored spores were then sampled over time to assay the number of colony-forming units (CFUs) on rich media (YEAS), which were normalized to the time zero starting point. In (A), three replicates are shown for each storage medium. In (B), The

bioRxiv preprint doi: https://doi.org/10.1101/2025.05.29.656811; this version posted May 30, 2025. The copyright holder for this preprint (which was not certified by peer review) is the author/funder, who has granted bioRxiv a license to display the preprint in perpetuity. It is made available under aCC-BY 4.0 International license.

average of 3 replicates is shown and the error bars represent standard deviations. ** indicates p value < 0.01, * indicates p value < 0.05, comparing areas under the curves via t-test. (C-G) Imaging analyses of spores stored for 67 days at 4°C in either water or yeast extract solution before being plated on rich media to induce germination at 32°C. Cells were imaged every 10 minutes for 1,200 minutes. (C) Timelapse microscopy images of germinating spores. The time in minutes is displayed and the scale bars represent 10 μm. (D) Cell area (μm^2) and (E) cell aspect ratio (AR) during germination. The shaded area around each line represents standard error. In some instances, the standard error is thinner than the line. Note that these cell areas provided by the deep learning algorithm are slightly larger than the actual cell areas (see methods). (F) Time of first division (time when the AR of a cell exceeded 3). NS indicates not significant via ANOVA test. N>100 spores per sample. (G) The fraction of spores manually observed to germinate by the end of the 1,200-minute timelapse. N>50 spores per sample. NS indicates not significant via Fisher's exact test.

Figure S11. Storage media significantly affects spore longevity, but not health. Imaging analyses of spores stored for 7 days at 42°C in either water or 0.5% yeast extract solution without glucose (YE) before being plated on rich media (YEAS) to induce germination at 32°C. Cells were imaged every 10 minutes for 1,200 minutes. (A) Timelapse microscopy images of germinating spores. The time in minutes is displayed and the scale bars represent 10 μm. (B) Cell area (μm^2) and (C) cell aspect ratio (AR) during germination. The shaded area around each line represents standard error. In some instances, the standard error is thinner than the line. Note that these cell areas provided by the deep learning algorithm are slightly larger than the actual cell areas (see methods). (D) Time of first division (time when the AR of a cell exceeded 3). NS indicates not significant via ANOVA test. N>100 spores per sample. (E) The fraction of spores manually observed to germinate by the end of the 1,200-minute timelapse. N>50 spores per sample. NS indicates not significant via Fisher's exact test.

bioRxiv preprint doi: https://doi.org/10.1101/2025.05.29.656811; this version posted May 30, 2025. The copyright holder for this preprint (which was not certified by peer review) is the author/funder, who has granted bioRxiv a license to display the preprint in perpetuity. It is made available under aCC-BY 4.0 International license.

Figure S12. Storage media significantly affects spore longevity, but not health. Imaging analyses of spores stored for 58 days at 4°C in either water or 0.5% yeast extract solution without glucose (YE) before being plated on rich media (YEAS) to induce germination at 37°C. Cells were imaged every 10 minutes for 1,200 minutes. (A) Timelapse microscopy images of germinating spores. The time in minutes is displayed and the scale bars represent 10 μm. (B) Cell area (μm^2) and (C) cell aspect ratio (AR) during germination. The shaded area around each line represents standard error. In some instances, the standard error is thinner than the line. Note that these cell areas provided by the deep learning algorithm are slightly larger than the actual cell areas (see methods). (D) Time of first division (time when the AR of a cell exceeded 3). NS indicates not significant via ANOVA test. N>100 spores per sample. (E) The fraction of spores manually observed to germinate by the end of the 1,200-minute timelapse. N>50 spores per sample. NS indicates not significant via Fisher's exact test. (F) Average time of first division and (G) Average germination efficiency of spores stored for 7-67 days in water (orange) or YE (green) at 4°C across all timelapses, including various germination temperatures and media (N=4).

Figure S13. Age affects spore health. (A) Imaging analyses of spores stored for 3, 157, or 282 days at 25°C in water before being plated on rich media (YEAS) to induce germination at 32°C. Cells were imaged every 10 minutes for 1,200 minutes. (A) Timelapse microscopy images of germinating spores. The time in minutes is displayed and the scale bars represent 10 μm. (B) Cell area (μm^2) and (C) aspect ratio (AR) during germination. The shaded area around each line represents standard error. In some instances, the standard error is thinner than the line. Note that these cell areas provided by the deep learning algorithm are slightly larger than the actual cell areas (see methods). (D) The fraction of spores manually observed to germinate by the end of the 1,200-minute timelapse. N>50 spores per sample. ** indicates p-value < 0.01 via Fisher's

bioRxiv preprint doi: https://doi.org/10.1101/2025.05.29.656811; this version posted May 30, 2025. The copyright holder for this preprint (which was not certified by peer review) is the author/funder, who has granted bioRxiv a license to display the preprint in perpetuity. It is made available under aCC-BY 4.0 International license.

exact test. (E) Time of first division (time when the AR of a cell exceeded 3). ** indicates p-value < 0.01 via ANOVA test. N>100 spores per sample. (F) The percentage of spores displaying the indicated outgrowth phenotypes. N>50 outgrowths per sample. ** indicates p-value < 0.01 via Fisher's exact test. (G) Starting area (μm^2) of spores. N>50 spores per sample. NS indicates not significant via ANOVA test.

Figure S14. Age affects spore health. Imaging analyses of spores stored for 7 or 176 days at 25°C in water before being plated on rich media (YEAS) in high (concentrated) or low (dilute) concentration to induce germination at 32°C. Cells were imaged every 10 minutes for 1,200 minutes. (A) Timelapse microscopy images of germinating spores. The time in minutes is displayed and the scale bars represent 10 μm. (B) Cell area (μm^2) and (C) aspect ratio (AR) during germination. The shaded area around each line represents standard error. In some instances, the standard error is thinner than the line. Note that these cell areas provided by the deep learning algorithm are slightly larger than the actual cell areas (see methods). (D) The fraction of spores manually observed to germinate by the end of the 1,200-minute timelapse. N>50 spores per sample. ** indicates p-value < 0.01 via Fisher's exact test. (E) Time of first division (time when the AR of a cell exceeded 3). ** indicates p-value < 0.01 via ANOVA test. N>100 spores per sample.

Figure S15. Age affects spore health. Imaging analyses of spores stored for 1 or 62 days at 25°C in water before being plated on synthetic media (PMG) to induce germination at 32°C. Cells were imaged every 10 minutes for 1,200 minutes. (A) Timelapse microscopy images of germinating spores. The time in minutes is displayed and the scale bars represent 10 μm. (B) Cell area (μm^2) and (C) aspect ratio (AR) during germination. The shaded area around each line represents standard error. In some instances, the standard error is thinner than the line. Note that these cell areas provided by the deep learning algorithm are slightly larger than the actual

bioRxiv preprint doi: https://doi.org/10.1101/2025.05.29.656811; this version posted May 30, 2025. The copyright holder for this preprint (which was not certified by peer review) is the author/funder, who has granted bioRxiv a license to display the preprint in perpetuity. It is made available under aCC-BY 4.0 International license.

cell areas (see methods). (D) The fraction of spores manually observed to germinate by the end of the 1,200-minute timelapse. N>50 spores per sample. ** indicates p-value < 0.01 via Fisher's exact test. (E) Time of first division (time when the AR of a cell exceeded 3). ** indicates p-value < 0.01 via ANOVA test. N>100 spores per sample.

Figure S16. Age affects spore health. Imaging analyses of spores stored for 1 or 85 days at 25°C in water before being plated on rich media (YEAS) to induce germination at 37°C. Cells were imaged every 10 minutes for 1,200 minutes. (A) Timelapse microscopy images of germinating spores. The time in minutes is displayed and the scale bars represent 10 μm. (B) Cell area (μm^2) and (C) aspect ratio (AR) during germination. The shaded area around each line represents standard error. In some instances, the standard error is thinner than the line. Note that these cell areas provided by the deep learning algorithm are slightly larger than the actual cell areas (see methods). (D) The fraction of spores manually observed to germinate by the end of the 1,200-minute timelapse. N>50 spores per sample. ** indicates p-value < 0.01 via Fisher's exact test. (E) Time of first division (time when the AR of a cell exceeded 3). ** indicates p-value < 0.01 via ANOVA test. N>100 spores per sample. (F) Average time of first division and (G) Average germination efficiency on YEAS of spores stored for 1-20, 30-100, or 150-240 days in water at 25°C across all timelapses, including various germination temperatures and media (N=4).

Figure S17. Age affects asymmetry of vacuole segregation during germination. Imaging analyses of vacuole segregation in FM4-64-stained spores previously stored at 25°C in water and then germinated on rich media (YEAS) at 32°C. The cells were imaged every 10 minutes for 20 hours. (A, C) Timelapse microscopy images of FM4-64-stained spores aged for 1 or 34 days (A) and 3 or 169 days (C). The brightness and contrast are not the same for all images but were adjusted so the spore bodies appeared to have similar levels of signal. Scale bars

bioRxiv preprint doi: https://doi.org/10.1101/2025.05.29.656811; this version posted May 30, 2025. The copyright holder for this preprint (which was not certified by peer review) is the author/funder, who has granted bioRxiv a license to display the preprint in perpetuity. It is made available under aCC-BY 4.0 International license.

represent 10 μm. (B, D) Percentage of the total FM4-64 signal retained in the spore-body verses the germ tube outgrowth for spores previously aged for 1 or 34 days (B) and 3 or 169 days (D). ** indicates p-value <0.01 via Fisher's exact test. N> 30 spores.

Figure S18. Age affects asymmetry of vacuole segregation during germination. Imaging analyses of vacuole segregation in FM4-64-stained spores previously stored in water for 5 or 173 days at 25°C, or for 5 days at 42°C. (A) Timelapse microscopy images of FM4-64-stained spores germinating on rich media (YEAS) at 32°C. The brightness and contrast are not the same for all images but were adjusted so the spore bodies appeared to have similar levels of signal. Scale bars represent 10 μm. (B) Percentage of the total FM4-64 signal retained in the spore-body verses the germ tube outgrowth. ** indicates p-value <0.01 via Fisher's exact test. N> 30 spores.

Figure S19. Age and stress affect spore stress tolerance. (A) The colony forming units (CFUs) of spore samples previously stored for 15, 100, 200 or 250 days at 25°C or for 15 days 37°C were assayed by plating on rich media (YEAS) at 32°C both before and after a 2-hour heat shock of 55°C. (B) CFUs of spores samples previously stored for 30 or 210 days at 25°C or 37°C for 30 days before and after exposure to 2500 μJules UV. ** indicates p-value < 0.01 via t-test. N≥3 replicates.

bioRxiv preprint doi: https://doi.org/10.1101/2025.05.29.656811; this version posted May 30, 2025. The copyright holder for this preprint (which was not certified by peer review) is the author/funder, who has granted bioRxiv a license to display the preprint in perpetuity. It is made available under aCC-BY 4.0 International license.

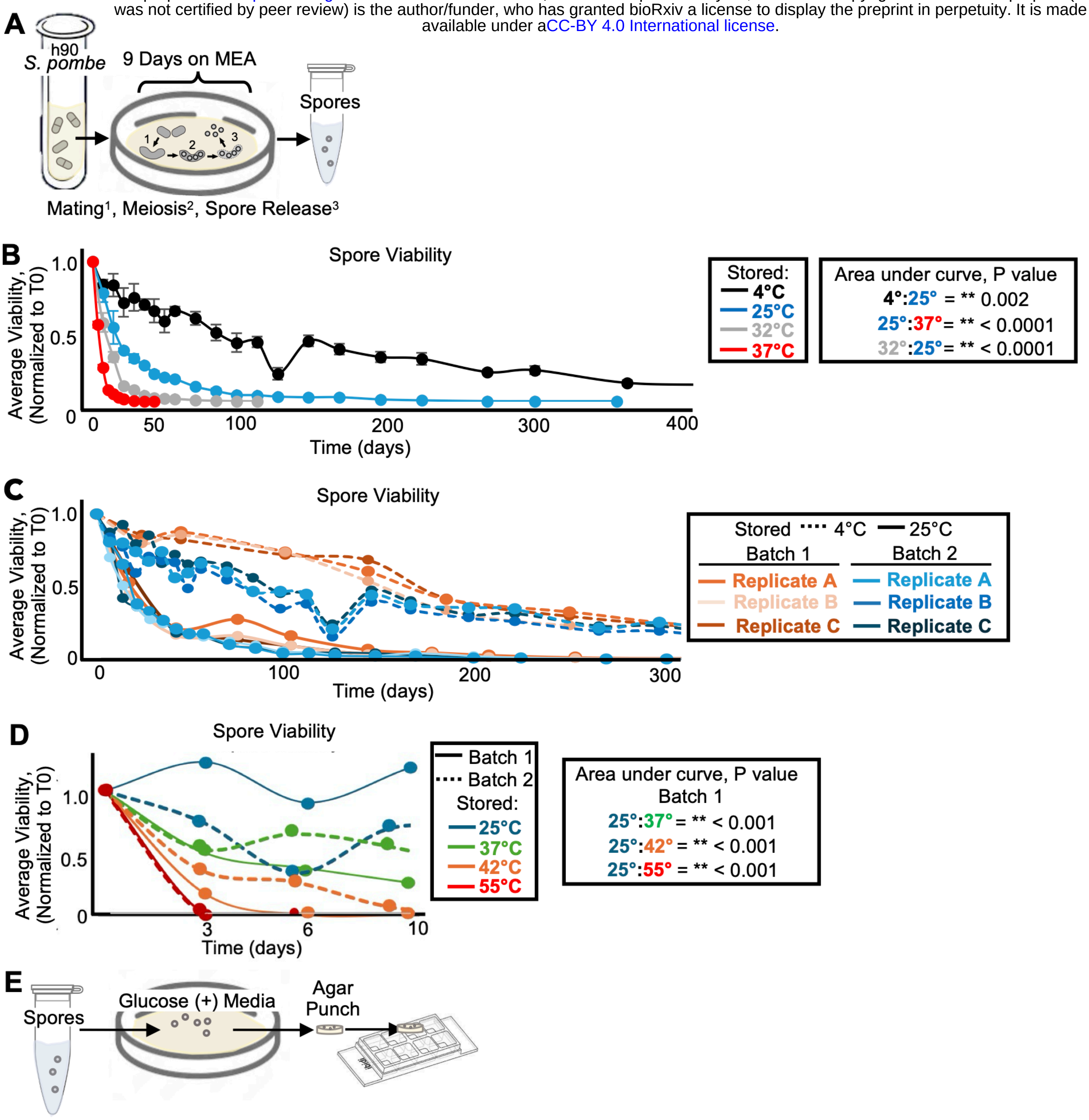

Figure S1

bioRxiv preprint doi: https://doi.org/10.1101/2025.05.29.656811; this version posted May 30, 2025. The copyright holder for this preprint (which was not certified by peer review) is the author/funder, who has granted bioRxiv a license to display the preprint in perpetuity. It is made available under aCC-BY 4.0 International license.

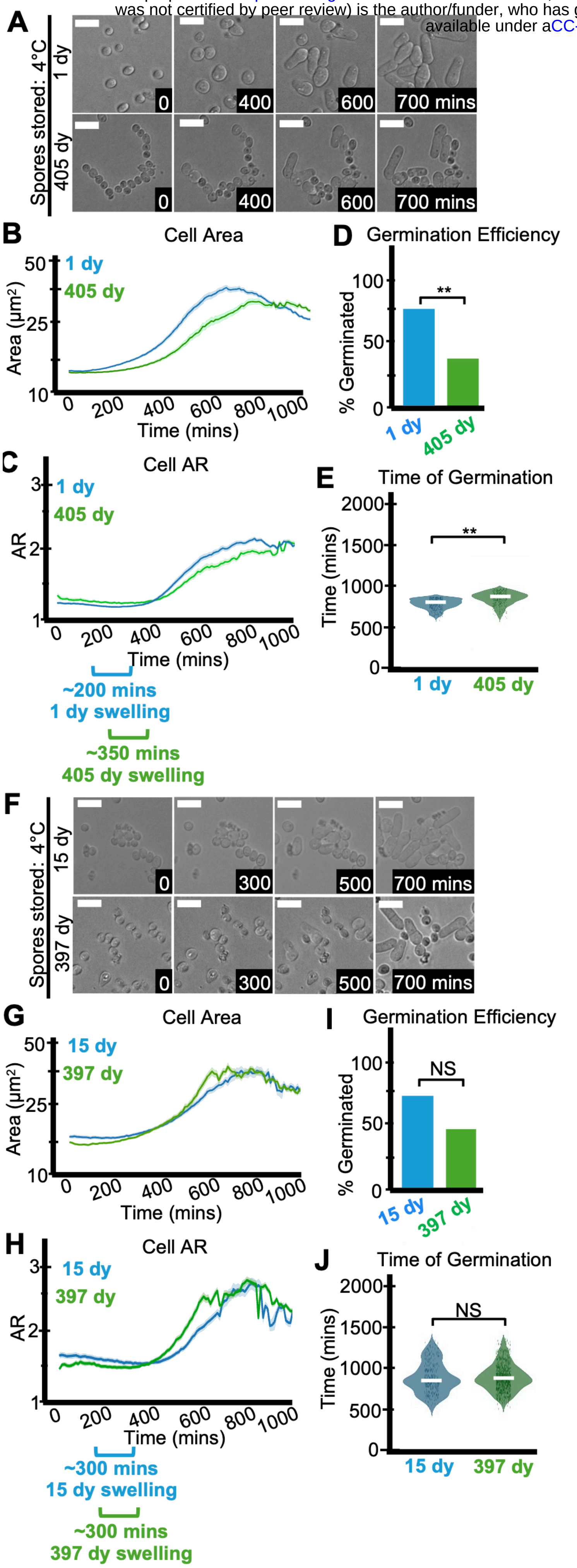

Figure S2

bioRxiv preprint doi: https://doi.org/10.1101/2025.05.29.656811; this version posted May 30, 2025. The copyright holder for this preprint (which was not certified by peer review) is the author/funder, who has granted bioRxiv a license to display the preprint in perpetuity. It is made available under aCC-BY 4.0 International license.

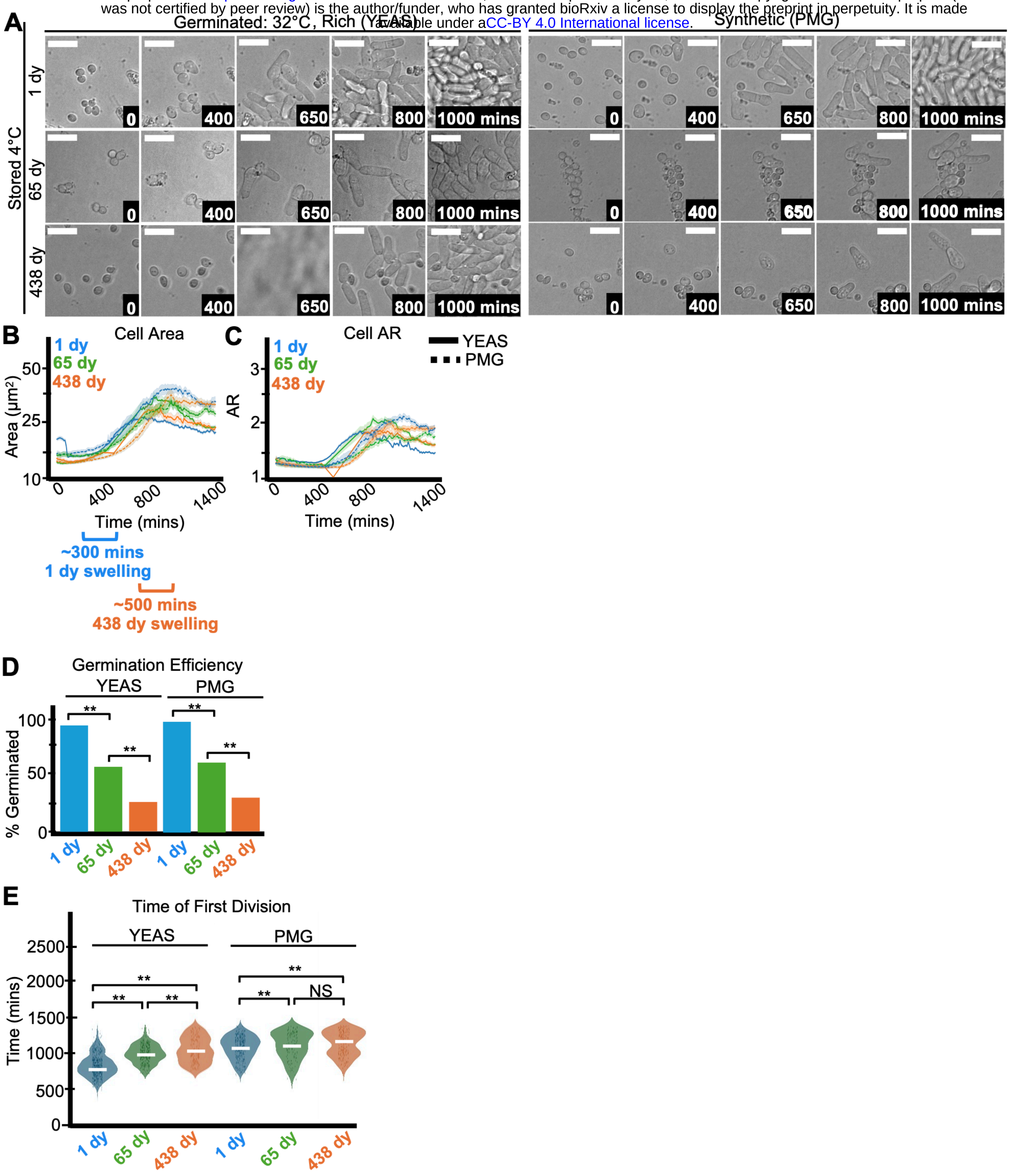

Figure S3

bioRxiv preprint doi: https://doi.org/10.1101/2025.05.29.656811; this version posted May 30, 2025. The copyright holder for this preprint (which was not certified by peer review) is the author/funder, who has granted bioRxiv a license to display the preprint in perpetuity. It is made available under aCC-BY 4.0 International license.

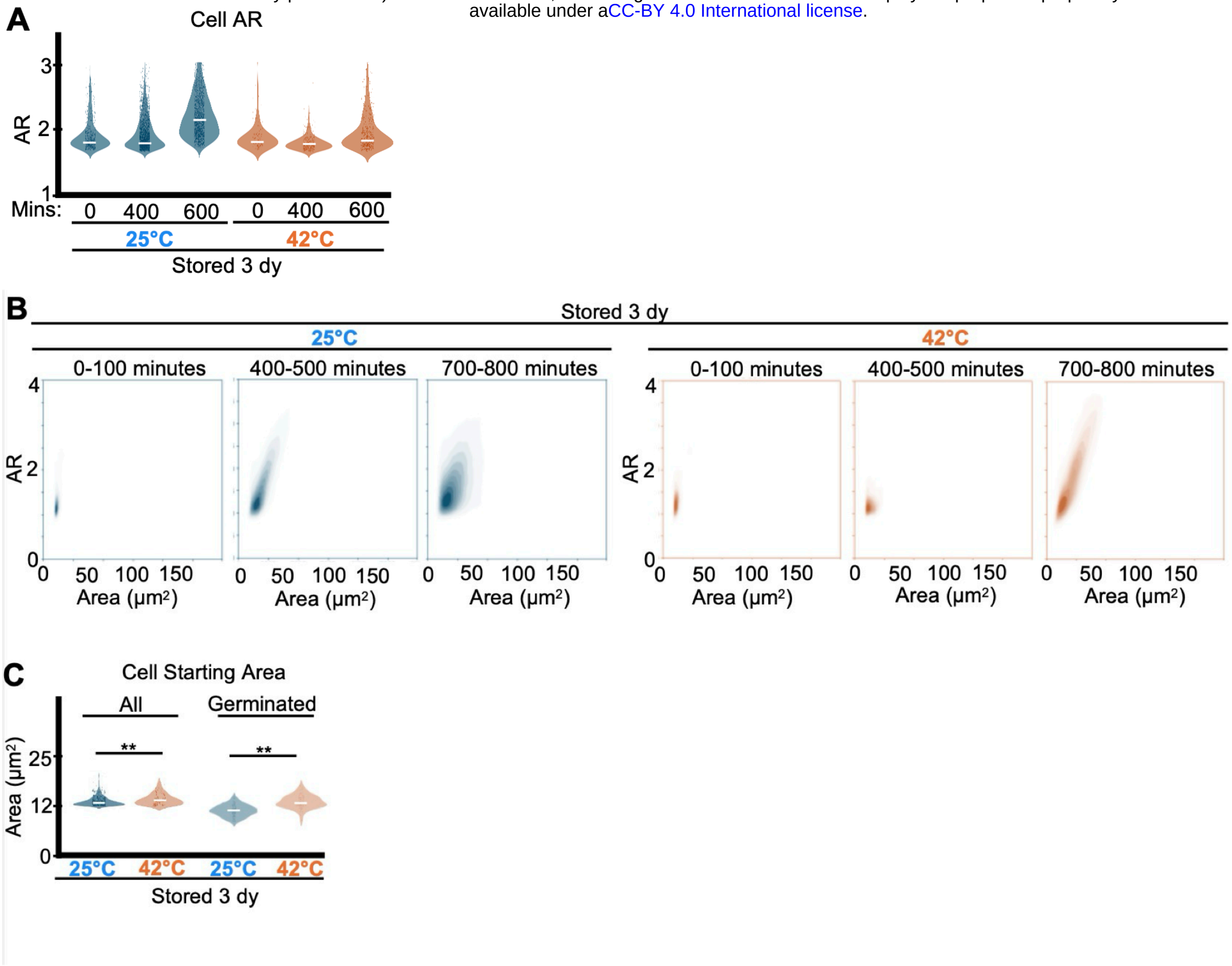

Figure S4

bioRxiv preprint doi: https://doi.org/10.1101/2025.05.29.656811; this version posted May 30, 2025. The copyright holder for this preprint (which was not certified by peer review) is the author/funder, who has granted bioRxiv a license to display the preprint in perpetuity. It is made available under aCC-BY 4.0 International license.

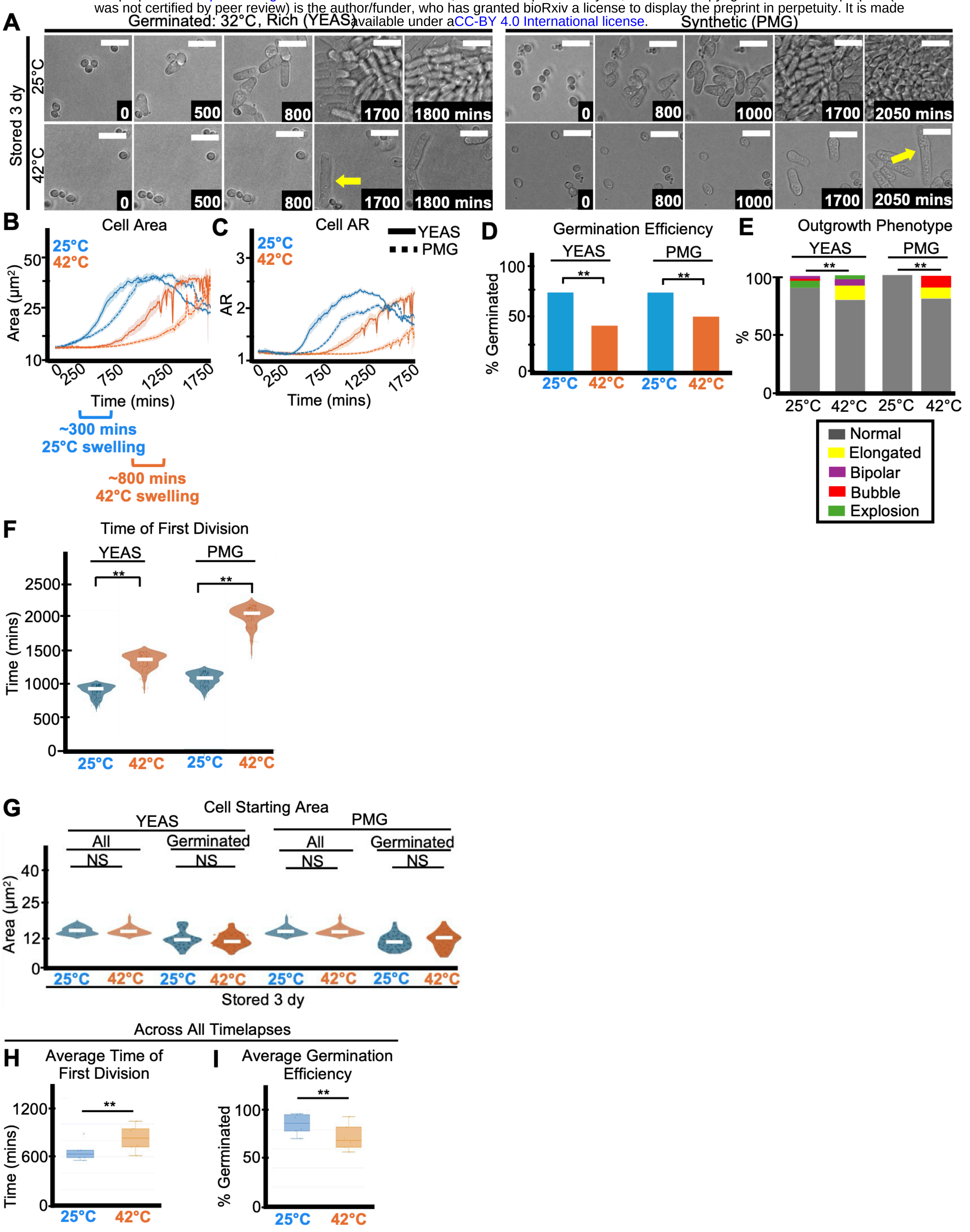

Figure S5

bioRxiv preprint doi: https://doi.org/10.1101/2025.05.29.656811; this version posted May 30, 2025. The copyright holder for this preprint (which was not certified by peer review) is the author/funder, who has granted bioRxiv a license to display the preprint in perpetuity. It is made available under aCC-BY 4.0 International license.

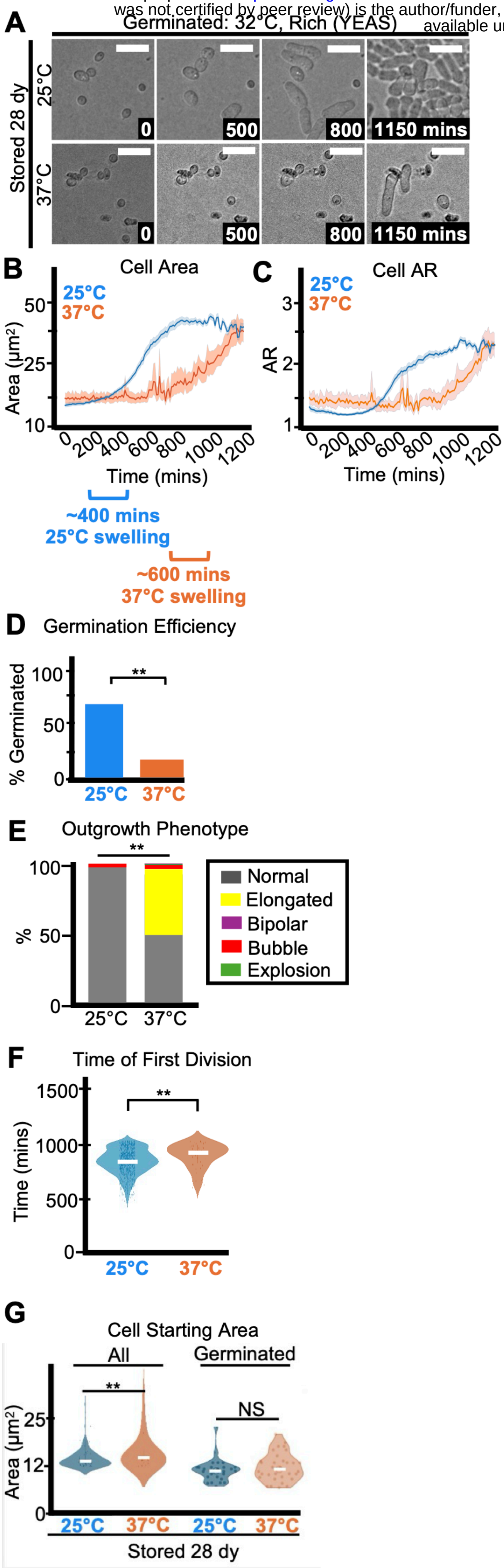

Figure S6

bioRxiv preprint doi: https://doi.org/10.1101/2025.05.29.656811; this version posted May 30, 2025. The copyright holder for this preprint (which was not certified by peer review) is the author/funder, who has granted bioRxiv a license to display the preprint in perpetuity. It is made available under aCC-BY 4.0 International license.

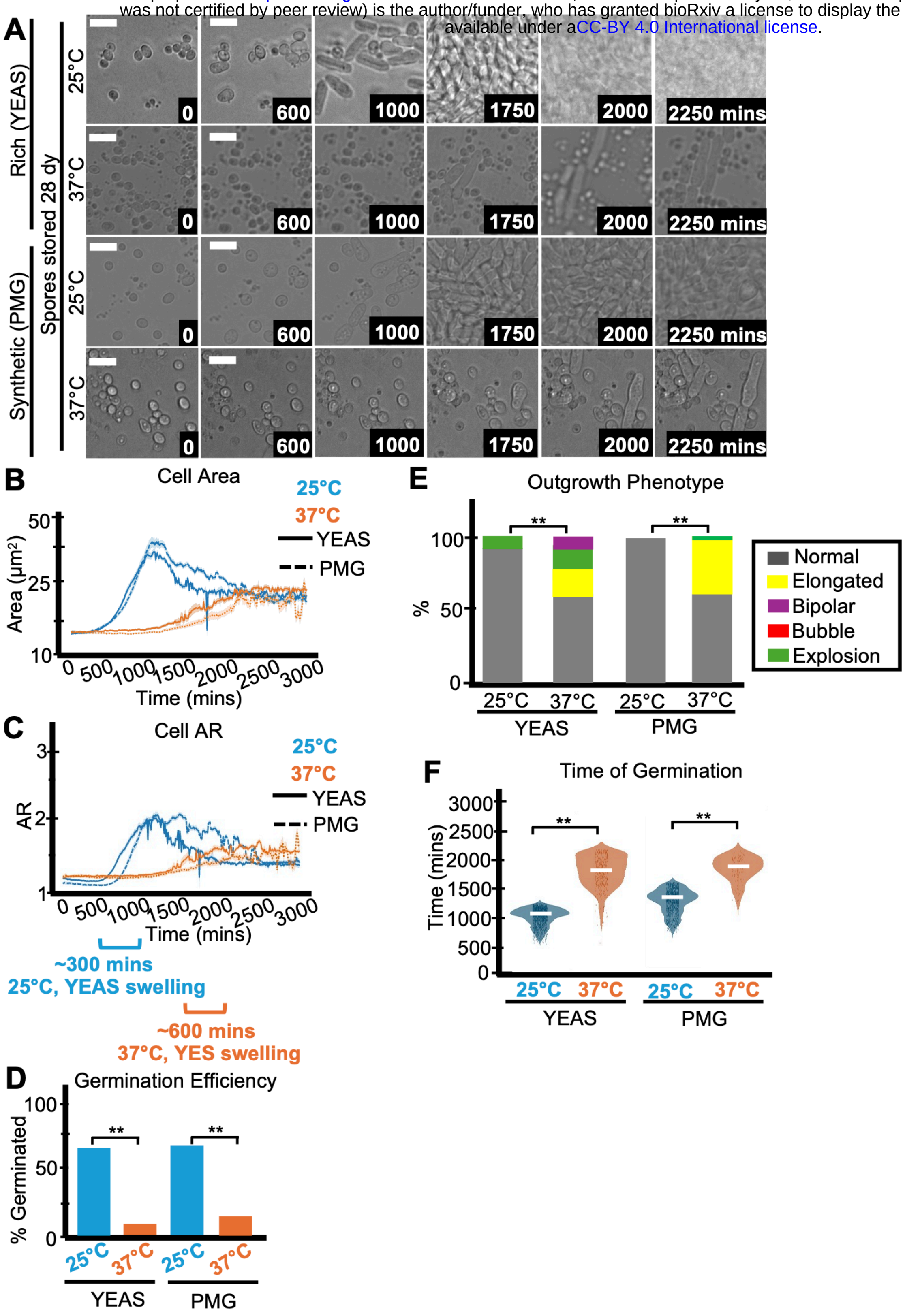

Figure S7

bioRxiv preprint doi: https://doi.org/10.1101/2025.05.29.656811; this version posted May 30, 2025. The copyright holder for this preprint (which was not certified by peer review) is the author/funder, who has granted bioRxiv a license to display the preprint in perpetuity. It is made available under aCC-BY 4.0 International license.

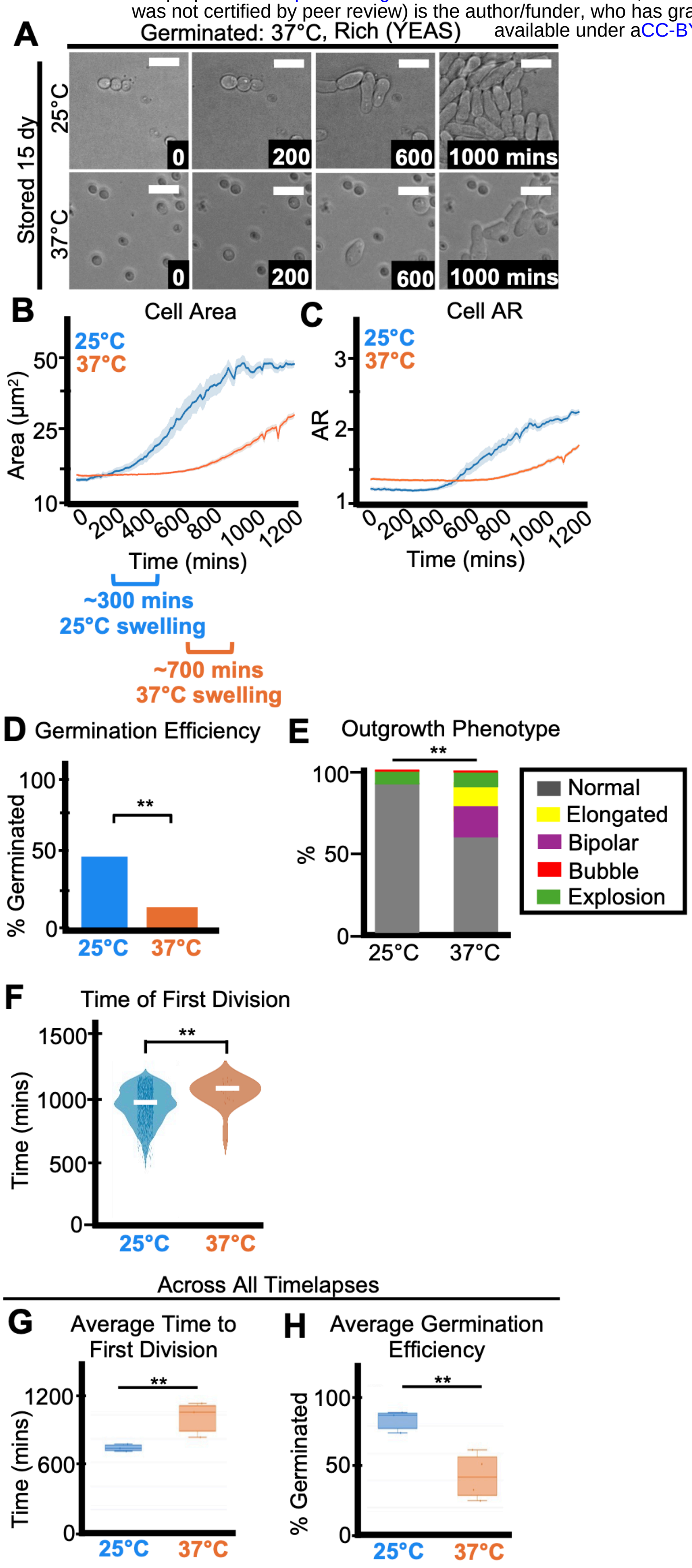

Figure S8

bioRxiv preprint doi: https://doi.org/10.1101/2025.05.29.656811; this version posted May 30, 2025. The copyright holder for this preprint (which was not certified by peer review) is the author/funder, who has granted bioRxiv a license to display the preprint in perpetuity. It is made available under aCC-BY 4.0 International license.

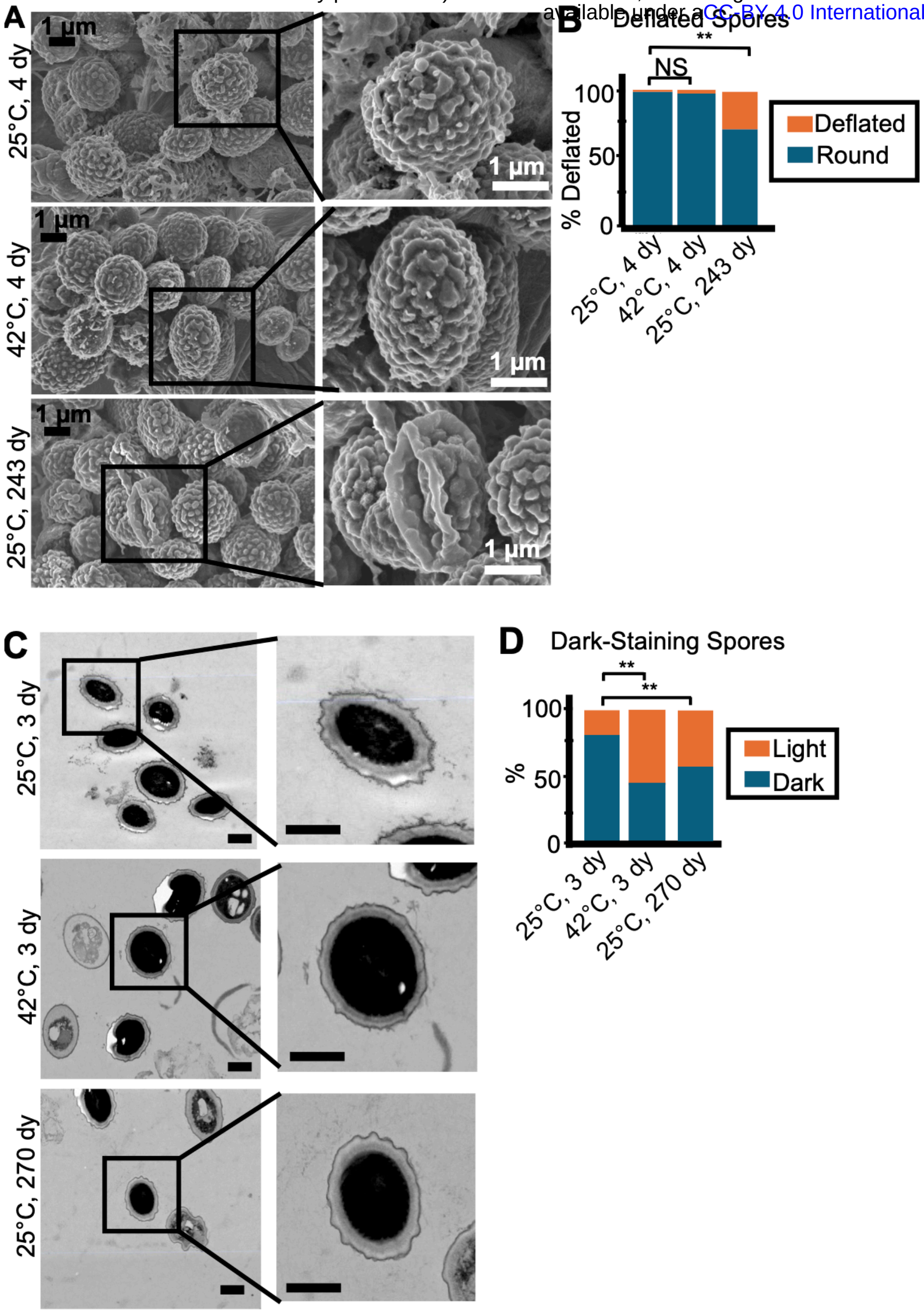

Figure S9

bioRxiv preprint doi: https://doi.org/10.1101/2025.05.29.656811; this version posted May 30, 2025. The copyright holder for this preprint (which was not certified by peer review) is the author/funder, who has granted bioRxiv a license to display the preprint in perpetuity. It is made available under aCC-BY 4.0 International license.

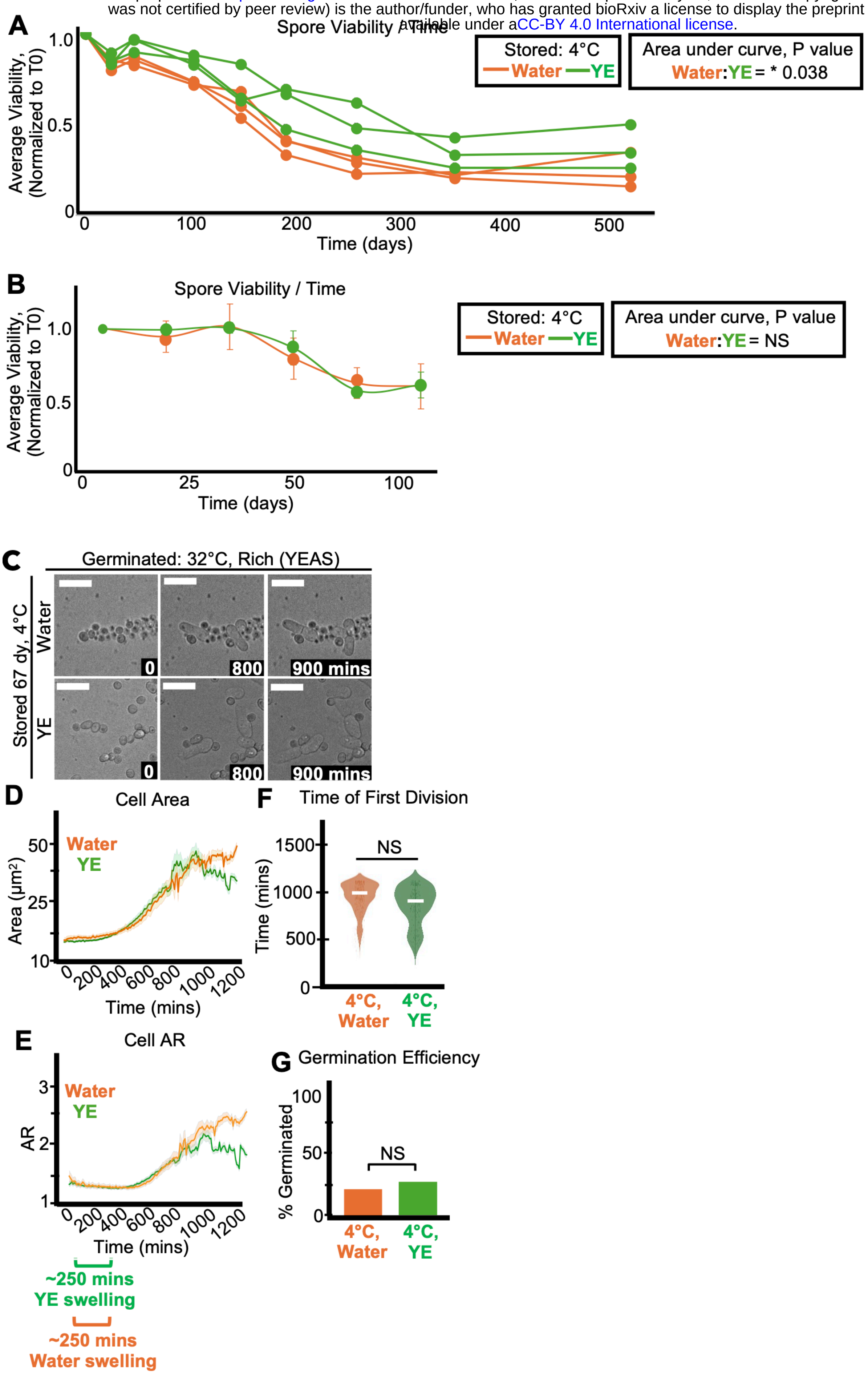

Figure S10

bioRxiv preprint doi: https://doi.org/10.1101/2025.05.29.656811; this version posted May 30, 2025. The copyright holder for this preprint (which was not certified by peer review) is the author/funder, who has granted bioRxiv a license to display the preprint in perpetuity. It is made available under aCC-BY 4.0 International license.

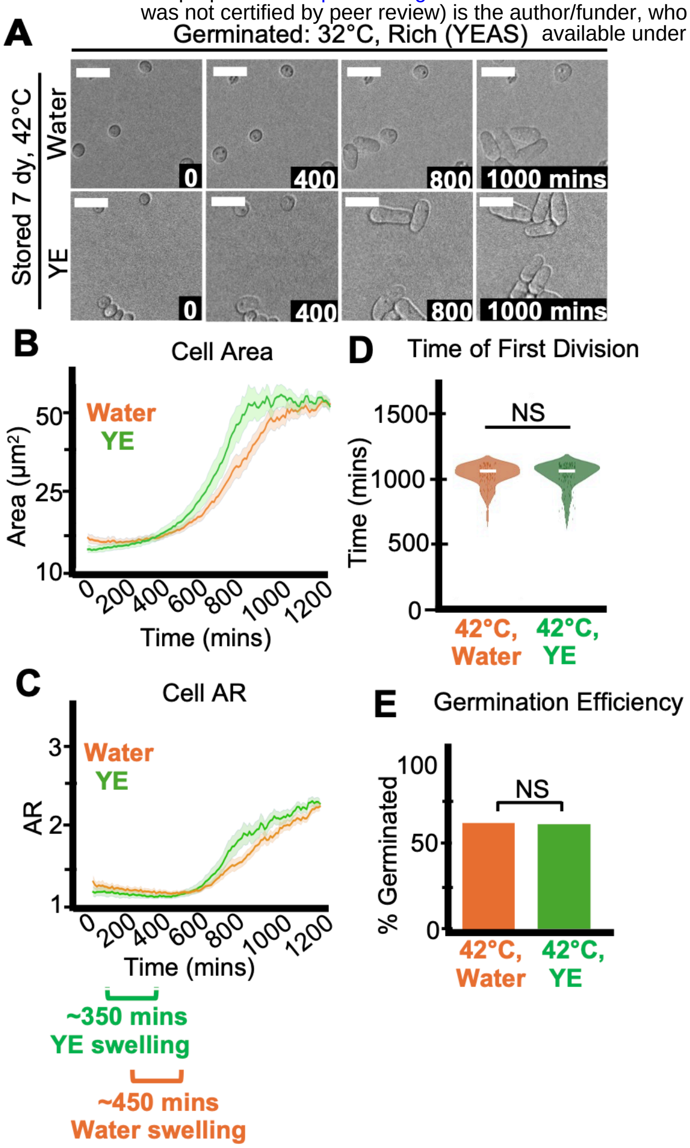

Figure S11

bioRxiv preprint doi: https://doi.org/10.1101/2025.05.29.656811; this version posted May 30, 2025. The copyright holder for this preprint (which was not certified by peer review) is the author/funder, who has granted bioRxiv a license to display the preprint in perpetuity. It is made available under aCC-BY 4.0 International license.

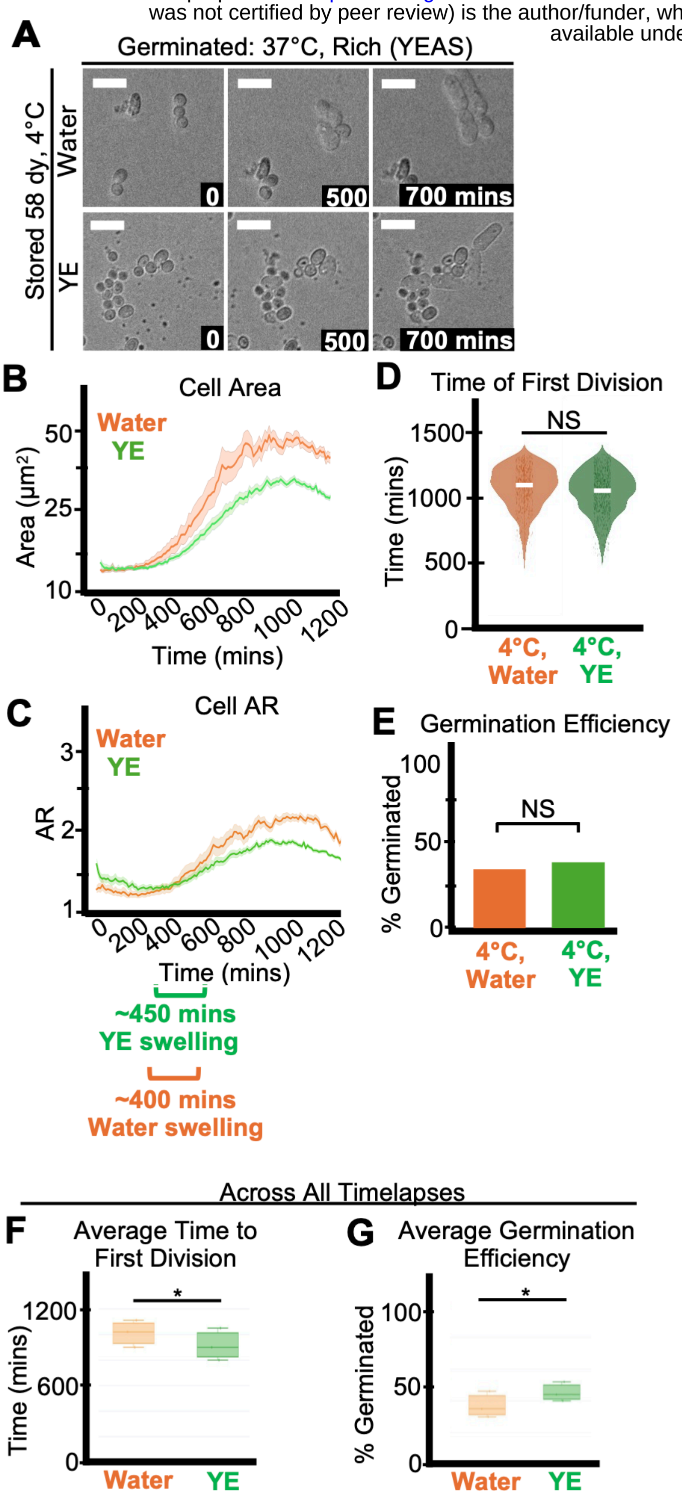

Figure S12

bioRxiv preprint doi: https://doi.org/10.1101/2025.05.29.656811; this version posted May 30, 2025. The copyright holder for this preprint (which was not certified by peer review) is the author/funder, who has granted bioRxiv a license to display the preprint in perpetuity. It is made available under aCC-BY 4.0 International license.

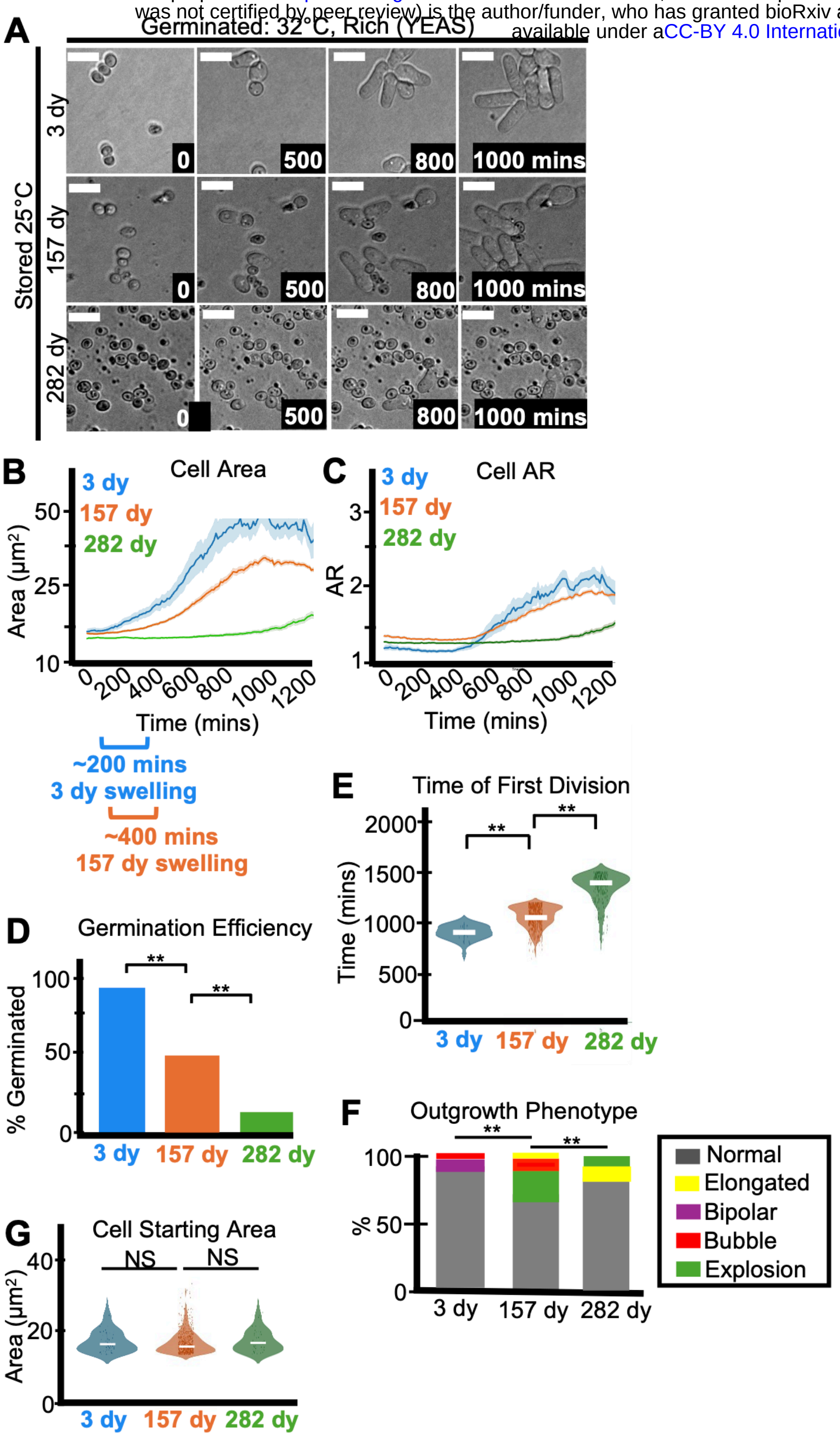

Figure S13

bioRxiv preprint doi: https://doi.org/10.1101/2025.05.29.656811; this version posted May 30, 2025. The copyright holder for this preprint (which was not certified by peer review) is the author/funder, who has granted bioRxiv a license to display the preprint in perpetuity. It is made available under aCC-BY 4.0 International license.

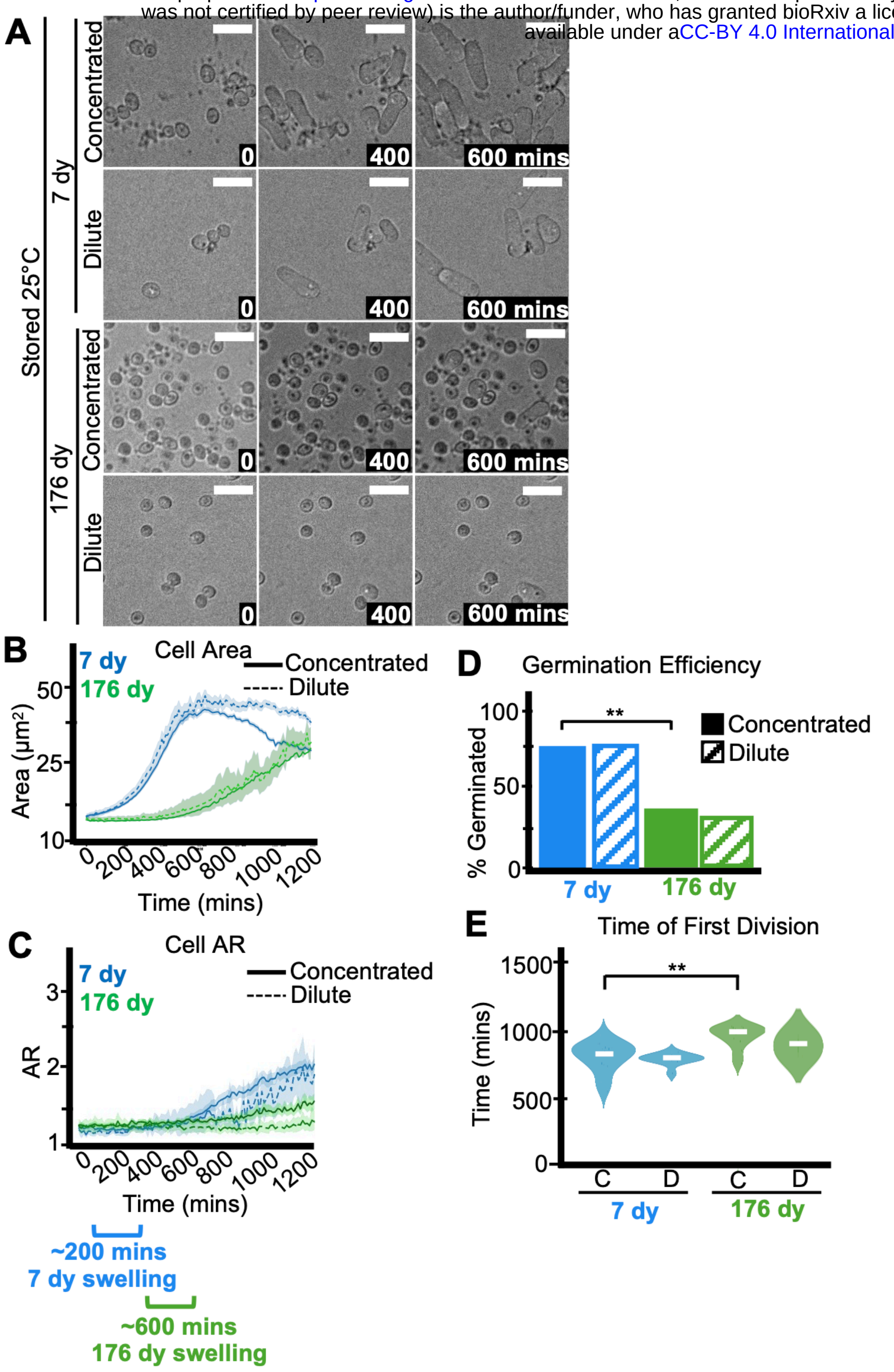

Figure S14

bioRxiv preprint doi: https://doi.org/10.1101/2025.05.29.656811; this version posted May 30, 2025. The copyright holder for this preprint (which was not certified by peer review) is the author/funder, who has granted bioRxiv a license to display the preprint in perpetuity. It is made available under aCC-BY 4.0 International license.

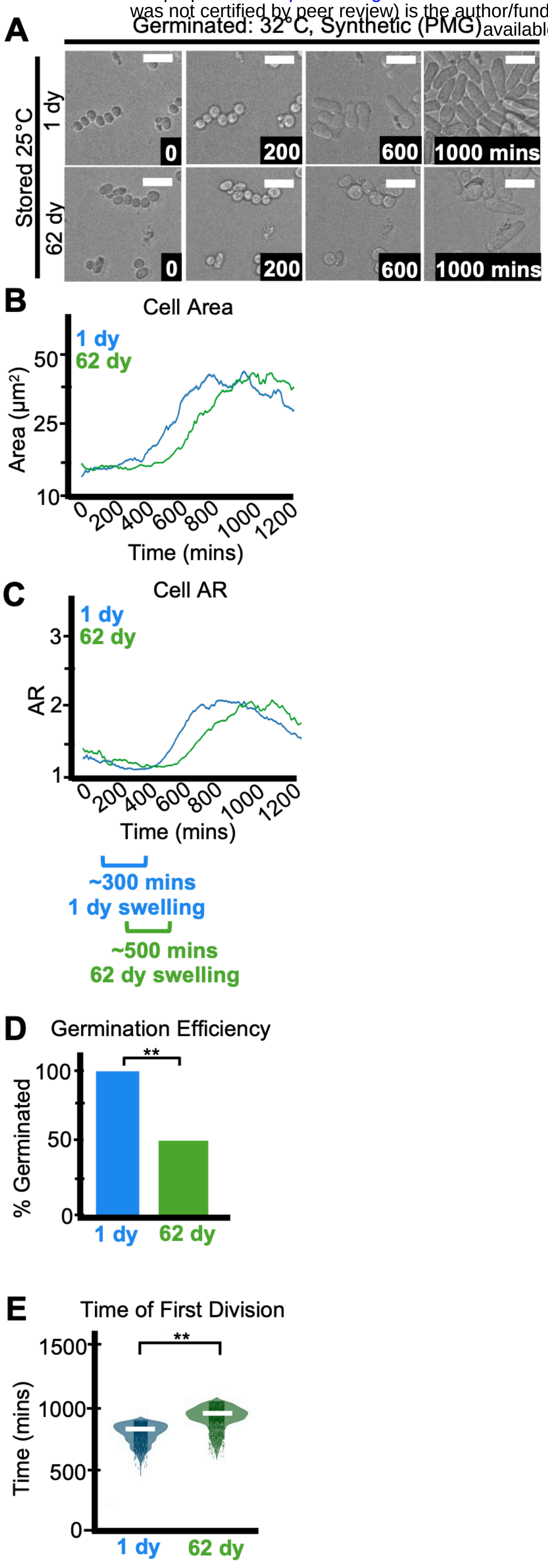

Figure S15

bioRxiv preprint doi: https://doi.org/10.1101/2025.05.29.656811; this version posted May 30, 2025. The copyright holder for this preprint (which was not certified by peer review) is the author/funder, who has granted bioRxiv a license to display the preprint in perpetuity. It is made available under aCC-BY 4.0 International license.

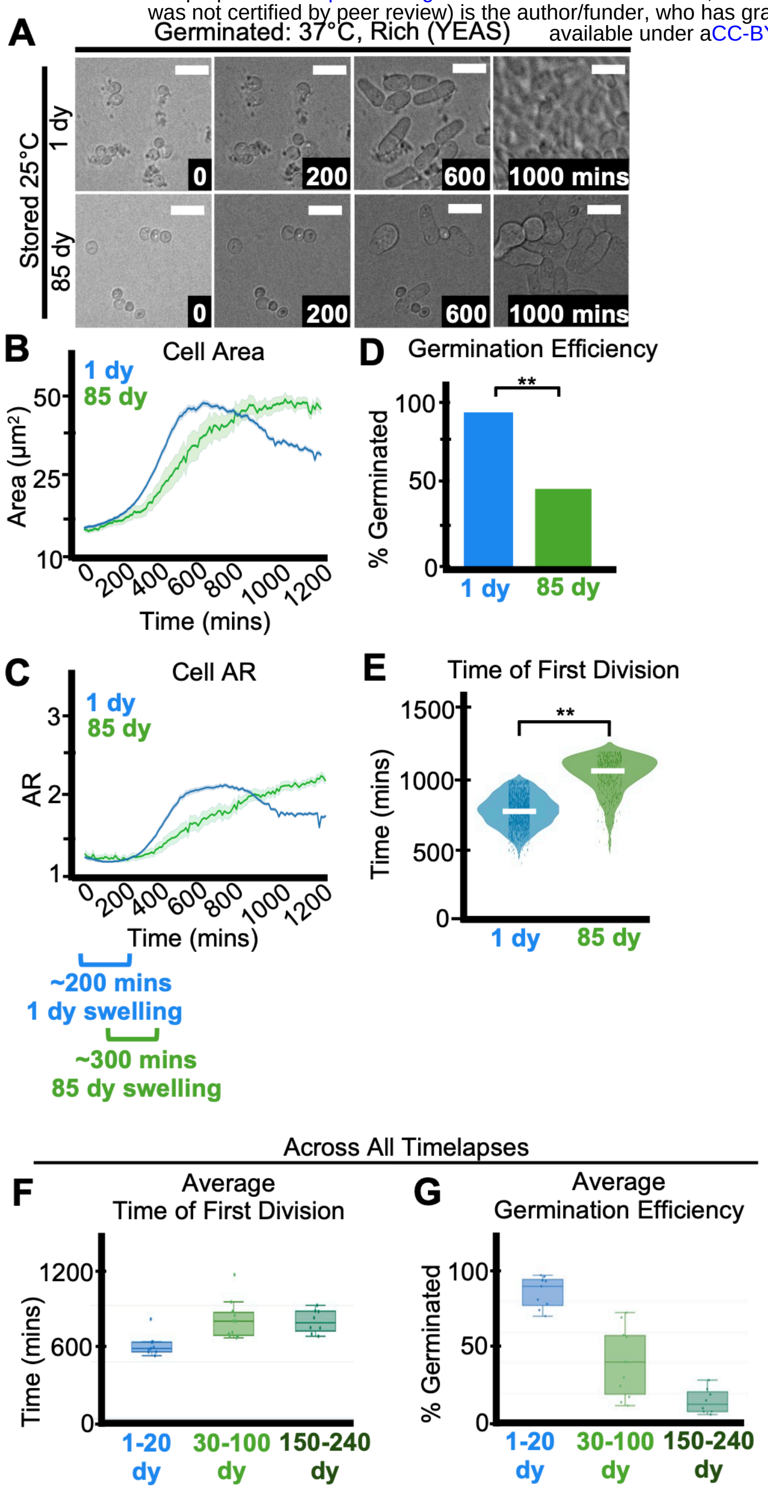

Figure S16

bioRxiv preprint doi: https://doi.org/10.1101/2025.05.29.656811; this version posted May 30, 2025. The copyright holder for this preprint (which was not certified by peer review) is the author/funder, who has granted bioRxiv a license to display the preprint in perpetuity. It is made available under aCC-BY 4.0 International license.

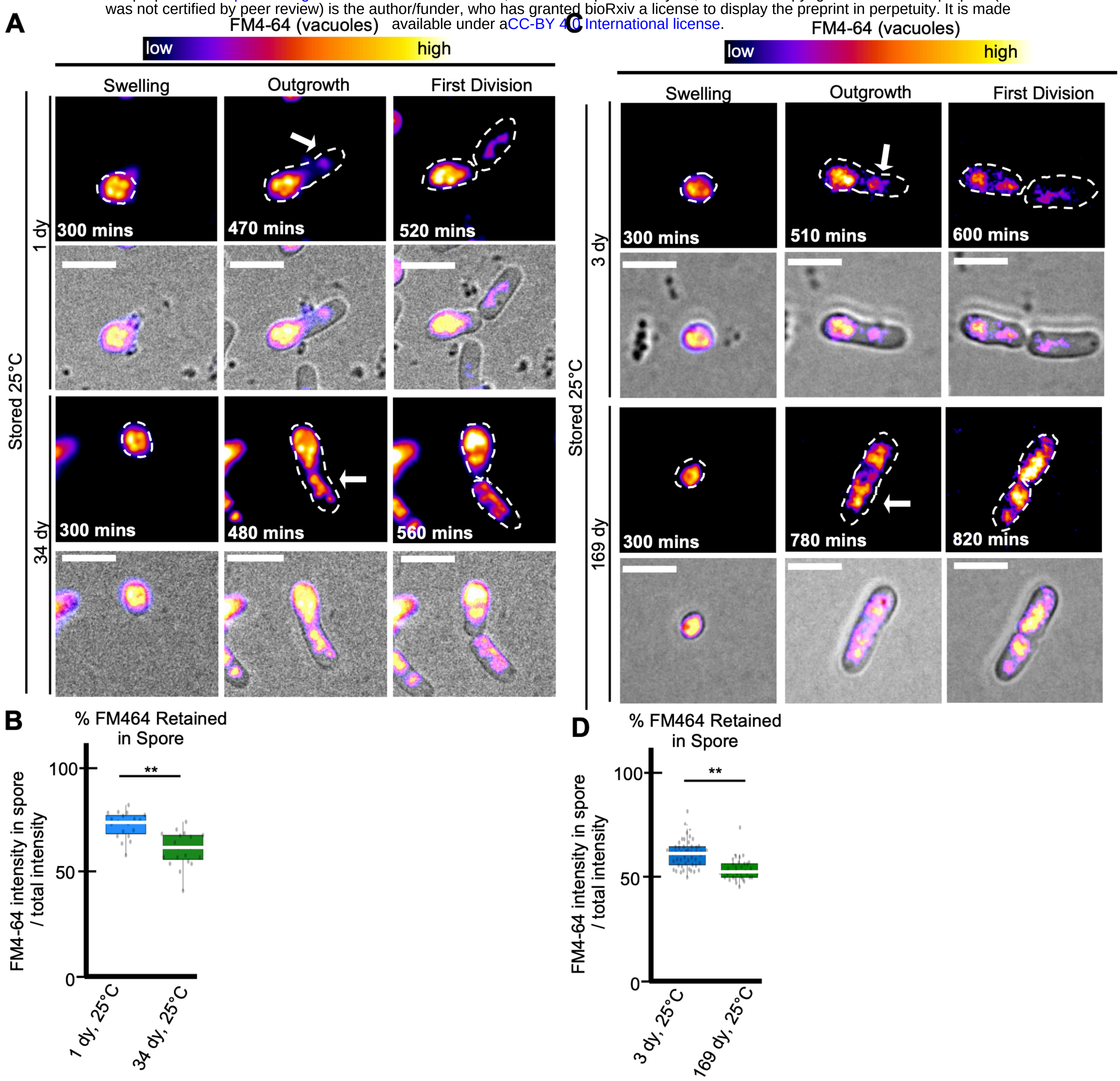

Figure S17

bioRxiv preprint doi: https://doi.org/10.1101/2025.05.29.656811; this version posted May 30, 2025. The copyright holder for this preprint (which was not certified by peer review) is the author/funder, who has granted bioRxiv a license to display the preprint in perpetuity. It is made available under aCC-BY 4.0 International license.

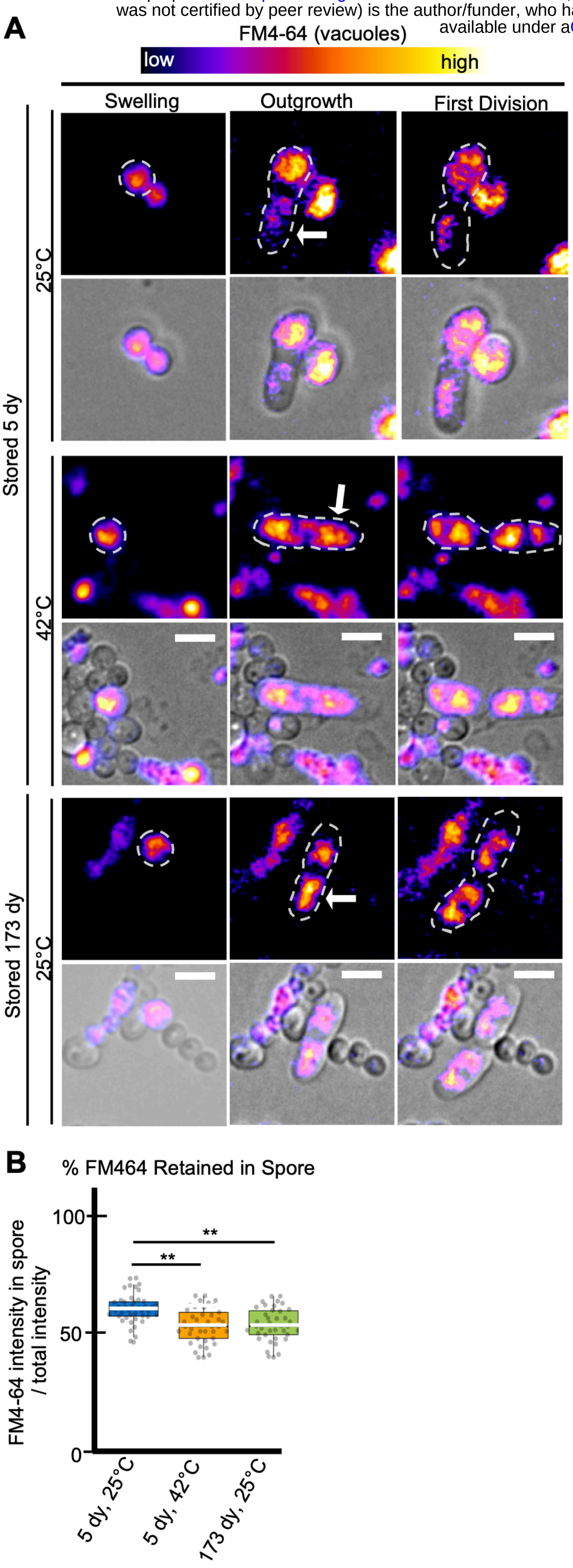

Figure S18

bioRxiv preprint doi: https://doi.org/10.1101/2025.05.29.656811; this version posted May 30, 2025. The copyright holder for this preprint (which was not certified by peer review) is the author/funder, who has granted bioRxiv a license to display the preprint in perpetuity. It is made available under aCC-BY 4.0 International license.

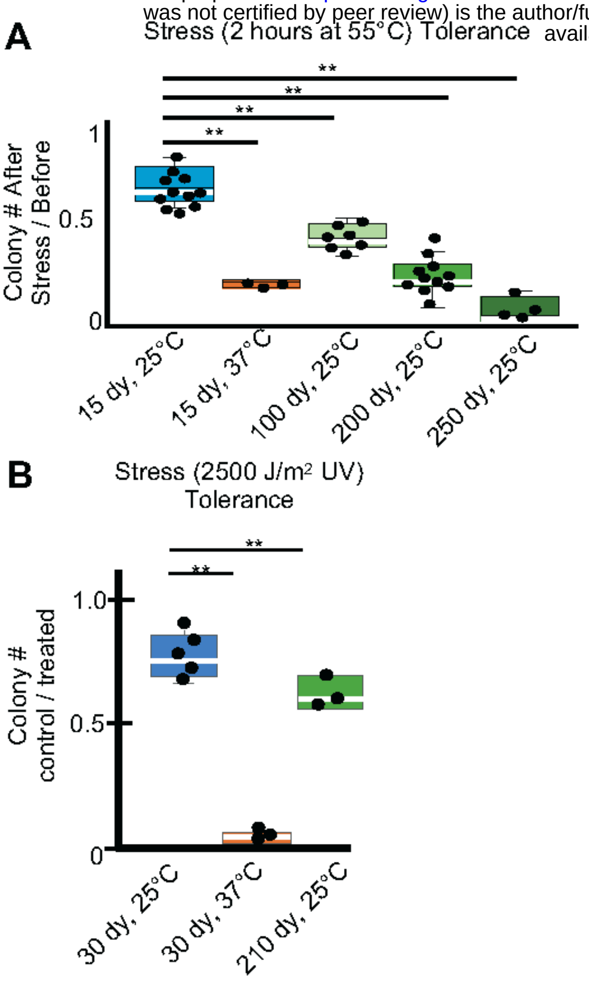

Figure S19
